# Retrospective analysis of individual risk factors for urethrocutaneous fistula after onlay hypospadias repair in pediatric patients

**DOI:** 10.1186/s13052-015-0140-8

**Published:** 2015-04-24

**Authors:** Li-Qu Huang, Zheng Ge, Jun Tian, Geng Ma, Ru-Gang Lu, Yong-Ji Deng, Li-Xia Wang, Chen-Jun Chen, Hao-Bo Zhu, Xiao-Jiang Zhu, Yun-Fei Guo

**Affiliations:** Department of Urology, Nanjing Children’s Hospital Affiliated to Nanjing Medical University, Nanjing, Jiangsu 210008 China; Department of Urology, Beijing Children’s Hospital Affiliated Hospital of Capital Medical University, Beijing, 100045 China

**Keywords:** Hypospadias, Urethrocutaneous fistula, Risk factors, Onlay island flap

## Abstract

**Background:**

To retrospectively identify the individual risk factors for the urethrocutaneous fistula (UCF) in pediatric patients after hypospadias repair (HR) with onlay island flap urethroplasty.

**Methods:**

A total of 167 patients who underwent primary HR at Nanjing Medical University Affiliated Children Hospital from January 2009 to December 2012 were enrolled. Clinical data including the patient’ age at HR, hypospadias type and urethral defect length were documented.

**Results:**

Among 167 patients, 12.6% patients (n = 21) developed UCF after HR. Postoperative UCF occurred in 3.9% (3/76) cases at age of 0–2 years, 14.3% (9/63) at 2–4 years, 20.0% (2/10) at 4–6 years and 38.9% (7/18) at 6–12 years. The incidences of UCF were 12.0% (3/25), 11.4% (5/132) and 30.0% (3/10) for distal, middle and proximal types of hypospadias. As to the urethral defect length, the incidences of UCF were 8.2% (5/61) in patients with a length of ≤ 2 cm, 12.8% (9/70) in 2-3 cm, 22.6% (7/31) in 3–4 cm and 0% (0/5) in above 4 cm. Older age at HR was significantly associated with the high incidence of UCF formation (*P* = 0.004), while the hypospadias type and urethral defect length did not affect it (*P* = 0.264 and *P* = 0.312, respectively).

**Conclusions:**

The patient’ age at HR was a risk factor for the UCF formation after HR, and treatment of HR within two years old might be with the least incidence of UCF.

## Background

Hypospadias is one of the most common urogenital anomalies with the prevalence rate of approximately 0.4 to 8.2 per 1000 live births [1,2]. Recent studies have shown a worldwide increasing incidence of hypospadias [2-4].

Surgical repair is the only treatment for hypospadias, which is as also called hypospadias repair (HR) [5,6]. Despite more than 100 different types of HR operation [7], onlay island flap and tabularized incised plate urethroplasty (TIP) are the two most popular procedures of choice for HR [8]. Each technique has its proponents. Onlay HR is associated with low complication rates [9-11]. However, urethrocutaneous fistula (UCF) is still an important postoperative complications of onlay HR, and often occurs in 5%-10% of hypospadias cases [2,12,13], especially within one month after operation, affecting the surgical outcome [13,14]. Although there are several studies on the variables related to UCF after HR [13-17], data on the individual risk factors of pediatric patients developing UCF remain rare.

Hence, this study reviewed a total of 167 pediatric patients who underwent primary HR with onlay island flap urethroplasty to identify the individual risk factors for UCF after HR.

## Materials and methods

The retrospective study enrolled a total of 167 pediatric patients who underwent primary HR with onlay island flap urethroplasty at Nanjing Children’s Hospital Affiliated to Nanjing Medical University from January 2009 to December 2012. Patients with no penile curvature or with mild penile curvature during erection were included in this study. All the procedures were approved by the Regional Ethics Committee of our hospital.

All the patients were evaluated and treated by the same experienced pediatric urologist. The clinical data including pediatric patient’s age at HR, the type of hypospadias and urethral defect length were documented. Regarding of the pediatric patient’s age at operation, it was assigned into four grades: 0–2 years, 2–4 years, 4–6 years and 6–12 years. According to the preoperative evaluation of urethral opening positions, hypospadias was classified into three types: proximal (penoscrotal, scrotal and perineal), middle (penile) and distal (glandular, coronal and subcoronal). In our study, the urethral defect length for the pediatric patients with no penile curvature was directly measured, while the urethral defect length for the cases with mild penile curvature was measured after penile straightening operation, with 0.5 cm as unit of measure. The length of urethral defect was classified into four grades as well: ≤ 2 cm, 2–3 cm, 3–4 cm and > 4 cm. The HR procedure with onlay island flap urethroplasty was performed according to the published technique [18].

The follow-up in this study was conducted one month after the extraction of urethral catheter, and the pediatric patients returned visit by the same pediatric urologist to confirm the presence of UCF.

Data are expressed as percentage. The influence of age, types of hypospadias and length of urethral defect on the likelihood of UCF was compared using univariate logistic regression analysis (ENTER method). All statistical analyses were performed using the standard statistical package of SPSS version 11.5 (SPSS Inc., Chicago, IL, USA). *P* < 0.05 was considered statistically significant.

## Results

Among the total of 167 pediatric patients undergoing initial HR, 21 (12.6%) pediatric patients developed UCF, while the remaining 146 patients were confirmed to be free of UCF (Table 1). Out of these 21 patients with documented UCF, 3 cases were less than two years old, 9 cases at the age of two-four years, 2 aged between four and six years, and 7 were more than six years old. The corresponding prevalence of UCF was 3.9%, 14.3%, 20.0% and 38.9%, respectively.

**Table 1:** **Characteristics of patients with or without UCF after HR**

**Characteristics**	**Patients (n)**	**UCF (−)**	**UCF (+)**
Total, n (%)	167	146 (87.4%)	21 (12.6%)
Age, n (%)			
0-2 years	76	73 (96.1%)	3 (3.9%)
2-4 years	63	54 (85.7%)	9 (14.3%)
4-6 years	10	8 (80%)	2 (20.0%)
6-12 years	18	11 (61.1%)	7 (38.9%)
Type of hypospadias			
Distal	25	22 (88%)	3 (12.0%)
Middle	132	117 (88.6%)	15 (11.4%)
Proximal	10	7 (70%)	3 (30.0%)
Urethral defect length			
≤ 2 cm	61	56 (91.8%)	5 (8.2%)
2-3 cm	70	61 (87.2%)	9 (12.8%)
3-4 cm	31	24 (77.4%)	7 (22.6%)
> 4 cm	5	5 (100%)	0

UCF: urethrocutaneous fistula; HR: hypospadias repair.

According to the preoperative evaluation, 25 pediatric patients have urethral opening at the distal position, 132 cases at the middle position, and 10 at the proximal position. Accordingly, the prevalence of UCF after HR were 12.0% (3/25), 11.4% (15/132) and 30.0% (3/10), respectively (Table 1).

As to the length of urethral defect, UCF occurred in 8.2% (5/61) patients with defect length less than 2 cm, 12.8% (9/70) cases with defect length of 2–3 cm and 22.6% (7/31) with defect length of 3–4 cm. However, the 5 patients with defect length > 4 cm did not developed UCF after HR (Table 1).

From the univariate analysis, the age was found to be a risk factor for developing UCF (Table 2, *P* = 0.004), especially at the ages between 2–4 years (OR = 4.056, *P* = 0.043) or above 6 years (OR = 15.485, *P* < 0.001). However, there was no relations of UCF with the hypospadias types or urethral defect length (*P* = 0.264 and *P* = 0.312, respectively).

**Table 2:** **Univariate analysis of risk factors for UCF after HR**

**Variable**	**Odds ratio**	**95% CI**	***P*** **- value**
Age (reference category: 0–2 years)			0.004
2-4 years	4.056	1.048-15.694	0.043
4-6 years	6.083	0.881-42.01	0.067
6-12 years	15.485	3.477-68.962	<0.001
Type of hypospadias (reference category: distal)			0.264
Middle	0.940	0.251-3.522	0.927
Proximal	3.143	0.513-19.248	0.216
Urethral defect length (reference category: ≤ 2 cm)			0.312
2-3 cm	1.652	0.522-5.228	0.393
3-4 cm	3.267	0.942-11.325	0.062
> 4 cm	0.00	0	0.999

UCF: urethrocutaneous fistula; HR: hypospadias repair.

An odds ratio > 1 with *P* < 0.05 indicates a greater likelihood of development of UCF, while an odds ratio < 1 with *P* < 0.05 indicates a lesser likelihood of development of UCF. An odds ratio = 1 with *P* < 0.05 indicates the given factor could not affect the development of UCF, but was significant in multivariate logistic regression model.

## Discussion

In spite of the accumulative experiences of surgeons and advanced surgical technique [19-21], however, there is still a high incidence of post-operative complications after HR, especially for UCF [22]. The management for complications after HR is a complex problem, no matter for the clinical practitioners, pediatricians and surgeons, but also for patients and their parents. In our investigation, UCF was confirmed in 12.6% (n = 21) out of the total pediatric patients after HR, which was consistent with the previously reported incidence of an estimated 5%-10% by other studies [2,12,13].

Several studies have focused on many risk factors associated with the complications after HR, such as hypospadias severity, availability of adequate tissue for reconstruction, surgeon’s experience and skill and whether there are previous failed attempts [6,14]. This study focused on the individual risk factors for the presence of UCF after HR. Pediatric patient’s age at HR was shown to significantly affect the postoperative incidence of UCF, and older children (> 6 years) with HR were more subjected to UCF. This was supported by previous research that reported age at operation was a risk factor for UCF in hypospadias surgery [23]. Thus in light of the results from this study and similar studies, it was recommend that hypospadias should be treated as earlier. The widely acknowledged optimal age to perform hypospadias repair was approximately between 6 and 12 months after birth recommend by American Academy of Pediatrics [2,7,24], during which time pediatric patients were not yet aware of the sexual identity with no erection [17]. Several potential explanations might account for it. First, erection was taken into consideration as evidenced by research on adult patient with hypospadias [13]. With the increasing age, erection occurred more frequently, and affected the postoperative complications notablely, especially for UCF [20]. In addition, it is widely known that the healing ability of younger children is stronger than older, which might be another reason for the lower incidence after successful surgical repair in younger patients. However, other studies also showed similar incidence of postoperative complications between pediatric patients with operation on younger than 8 months or older than 8 months [25].

Chung *et al.* reported that UCF formation after HR was significantly associated with the location of hypospadias [17]. Conversely, no statistically significant difference in incidence was seen between patients in terms of various types of hypospadias. The explanation for this finding was probably multifactorial. Most cases with hypospadias were associated with membranous urethra opening and usually with penile bending. During operation, it needed to open the membranous urethra for fully straightening the curved penis. Practically, it was found that the urethra open position moves backwards from the original site, meaning that the original urethral opening position cannot adequately reflect the type of hypospadias. Another potential explanation might be that the development of phallosome was not fully considered when evaluating the hypospadias type. With regard to mild hypospadias, patients with differently developed penis would cause different difficulty related to the operation, which might affect the formation of UCF after HR.

Few studies have focused on the relationship between the incidence of UCF after HR and urethral defect length. In our investigation, this parameter was taken into the consideration according to the following reason. First, the blood supply to anastomotic area with the skin flap and the original urethra was usually very poor [26]; Second, healing of two different kinds of tissues was relatively difficult; What’s more, most cases of UCF occurred in the anastomotic site of old and new urethra. However, the result did not show a relationship between this parameter and the presence of UCF as we have expected. The small cases of pediatric patients developing UCF and the short follow-up time might be responsible for it.

Several limitations to this study must be addressed. First, it was a case series study with data collected retrospectively. Second, the cases of pediatric patients who developed UCF after HR were not sufficient and the follow-up might be a little short, thus it might affect the statistical accuracy. Third, the individual risk factors were limited to some measured variables. Hence, further experimental research would be needed on a larger number of pediatric patients undergoing HR for a long-time follow up. However, the risk factor identified in our study was beneficial for surgeons and patients/parents when considering surgical treatment of hypospadias.

## Conclusions

This study supported that the patient’ age at HR was a significant risk factor for the presence of UCF after primary HR, whereas the type of the hypospadias and the urethral tract length were not associated with UCF formation.

## References

[1] Baskin LS, Ebbers MB (2006). Hypospadias: anatomy, etiology, and technique. J Pediatr Surg.

[2] Leung AK, Robson WL (2007). Hypospadias: an update. Asian J Androl.

[3] Dolk H (1998). Rise in prevalence of hypospadias. The Lancet.

[4] Paulozzi LJ (1999). International trends in rates of hypospadias and cryptorchidism. Environ Health Perspect.

[5] Mieusset R, Soulié M (2005). Hypospadias: psychosocial, sexual, and reproductive consequences in adult life. J Androl.

[6] Nuininga JE, De Gier RP, Verschuren R, Feitz WF (2005). Long-term outcome of different types of 1-stage hypospadias repair. J Urol.

[7] Manzoni G, Bracka A, Palminteri E, Marrocco G (2004). Hypospadias surgery: when, what and by whom?. BJU Int.

[8] Cook A, Khoury AE, Neville C, Bagli DJ, Farhat WA, Salle JLP (2005). A multicenter evaluation of technical preferences for primary hypospadias repair. J Urol.

[9] BARROSO U, Jednak R, Barthold JS, Gonzalez R (2000). Further experience with the double onlay preputial flap for hypospadias repair. J Urol.

[10] Chin T, Liu C, Wei C (2001). Hypospadias repair using a double onlay preputial flap. Pediatr Surg Int.

[11] Emir L, Germiyanoglu C, Erol D (2003). Onlay island flap urethroplasty: a comparative analysis of primary versus reoperative cases. Urology.

[12] Shankar KR, Losty PD, Hopper M, Wong L, Rickwood AM (2002). Outcome of hypospadias fistula repair. BJU Int.

[13] Wood HM, Kay R, Angermeier KW, Ross JH (2008). Timing of the Presentation of Urethrocutaneous Fistulas After Hypospadias Repair in Pediatric Patients. J Urol.

[14] Snyder CL, Evangelidis A, Hansen G, St. Peter SD, Ostlie DJ, Gatti JM, Gittes GK, Sharp RJ, Murphy JP (2005). Management of complications after hypospadias repair. Urology.

[15] Waterman BJ, Renschler T, Cartwright PC, Snow BW, de Vries CR (2002). Variables in Successful Repair of Urethrocutaneous Fistula After Hypospadias Surgery. J Urol.

[16] Barbagli G, Perovic S, Djinovic R, Sansalone S, Lazzeri M (2010). Retrospective Descriptive Analysis of 1,176 Patients With Failed Hypospadias Repair. J Urol.

[17] Chung JW, Choi SH, Kim BS, Chung SK (2012). Risk factors for the development of urethrocutaneous fistula after hypospadias repair: a retrospective study. Korean J urology.

[18] Elder J, Duckett J, Snyder H (1987). Onlay island flap in the repair of mid and distal penile hypospadias without chordee. J Urol.

[19] Cimador M, Castagnetti M, De Grazia E (2003). Urethrocutaneous fistula repair after hypospadias surgery. BJU Int.

[20] Latifoglu O, Yavuzer R, Unal S, Cavusoglu T, Atabay K (2000). Surgical treatment of urethral fistulas following hypospadias repair. Ann Plast Surg.

[21] Landau EH, Gofrit ON, Meretyk S, Katz G, Golijanin D, Shenfeld OZ, Pode D (2003). Outcome analysis of tunica vaginalis flap for the correction of recurrent urethrocutaneous fistula in children. J Urol.

[22] Demirbilek S, Kanmaz T, Aydin G, Yucesan S (2001). Outcomes of one-stage techniques for proximal hypospadias repair. Urology.

[23] Yildiz T, Tahtali IN, Ates DC, Keles I, Ilce Z (2013). Age of patient is a risk factor for urethrocutaneous fistula in hypospadias surgery. J Pediatr Urol.

[24] Kass E, Kogan SJ, Manley C, Wacksman JA, Klykylo WM, Meza A (1996). Timing of elective surgery on the genitalia of male children with particular reference to the risks, benefits, and psychological effects of surgery and anesthesia. Pediatrics.

[25] Weber DM, Schonbucher VB, Gobet R, Gerber A, Landolt MA (2009). Is there an ideal age for hypospadias repair? A pilot study. J Pediatr Urol.

[26] Dabernig J, Shelley OP, Cuccia G, Schaff J (2007). Urethral reconstruction using the radial forearm free flap: experience in oncologic cases and gender reassignment. Eur Urol.

